# Increased Iron Levels and Oxidative Stress Mediate Age-Related Impairments in Male and Female *Drosophila melanogaster*

**DOI:** 10.1155/2023/7222462

**Published:** 2023-06-09

**Authors:** Karen Kich Gomes, Ana Beatriz dos Santos, Jaciana Sousa dos Anjos, Luana Paganotto Leandro, Maria Takemura Mariano, Felipe Lima Pinheiro, Marcelo Farina, Jeferson Luis Franco, Thais Posser

**Affiliations:** ^1^Oxidative Stress and Cell Signaling Research Group, Interdisciplinary Research Center on Biotechnology-CIPBIOTEC, Universidade Federal do Pampa, Campus São Gabriel, RS, Brazil; ^2^Department of Chemistry, Post Graduate Program in Toxicological Biochemistry, Universidade Federal de Santa Maria, RS, Brazil; ^3^Paleontology Laboratory, Federal University of Pampa, Campus São Gabriel, RS, Brazil; ^4^Department of Biochemistry, Federal University of Santa Catarina, Florianopolis, 88040-900 Santa Catarina, Brazil

## Abstract

Aging is characterized by a functional decline in the physiological functions and organic systems, causing frailty, illness, and death. Ferroptosis is an iron- (Fe-) dependent regulated cell death, which has been implicated in the pathogenesis of several disorders, such as cardiovascular and neurological diseases. The present study investigated behavioral and oxidative stress parameters over the aging of *Drosophila melanogaster* that, together with augmented Fe levels, indicate the occurrence of ferroptosis. Our work demonstrated that older flies (30-day-old) of both sexes presented impaired locomotion and balance when compared with younger flies (5-day-old). Older flies also produced higher reactive oxygen species (ROS) levels, decreased glutathione levels (GSH), and increased lipid peroxidation. In parallel, Fe levels were augmented in the fly's hemolymph. The GSH depletion with diethyl maleate potentiated the behavioral damage associated with age. Our data demonstrated biochemical effects that characterize the occurrence of ferroptosis over the age of *D. melanogaster* and reports the involvement of GSH in the age-associated damages, which could be in part attributed to the augmented levels of Fe.

## 1. Introduction

The aging process is a physiological event commonly associated with neuronal loss, motor disabilities, metabolism alterations, cellular senescence, augmented reactive oxygen species (ROS) levels, lipid peroxidation, fragility, and increased susceptibility to diseases [1–3]. Harman and his theory of free radicals (1950) described the basal mechanism of aging as a result of the accumulation of ROS, produced as by-products of cellular metabolism and decreasing lifespan [4, 5]. More recent studies demonstrated that additional processes including Fe (Fe) accumulation are key players in accelerating aging [6].

Iron (Fe) is an important metal that participates in several biological processes in eukaryotes, including cell respiration and energy production, DNA synthesis, ecdysone, lipids, and dopamine metabolism [7, 8]. Considering the importance of this metal, alterations in its regulatory mechanism causing its accumulation or deficiency can have devastating consequences [9, 10]. When in excess, Fe can interact with hydrogen peroxide (H_2_O_2_) generating hydroxyl radicals (OH°) through the Fenton reaction, which plays critical oxidative damage to biomolecules and is associated with the onset of diseases like cancer, diabetes, and neurodegenerative and cardiovascular diseases [7, 10, 11].

Ferroptosis is a Fe-dependent cell death composed of four main stages: depletion of GSH and/or GPX4, increase in intracellular Fe, excessive production of ROS, and lipid peroxidation [12–14]. Characteristics of ferroptosis include the toxic end products of lipid peroxidation, 4-hydroxynonenal (4-HNE), and malondialdehyde (MDA), which are mutagenic and cytotoxic molecules [14]. In ferroptosis, cell death occurs due to Fe-dependent lipid peroxidation caused by an augmented ROS production [15], via Fenton reaction. A decrease in GSH-dependent antioxidant defenses also contributes to the ferroptosis process, since it compromises the activity of GSH-dependent hydroperoxidase that converts lipid peroxides into nontoxic lipid alcohols [12, 16, 17].

GSH is the most abundant cellular thiol in living beings, acting as a potent endogenous antioxidant. It plays important roles in Fe homeostasis, acting in Fe sensing and regulation, Fe trafficking, and Fe cofactor biosynthesis. Many GSH reactions involve the highly polarizable sulfhydryl (SH) group, making it a good nucleophile for reactions with electrophilic chemical compounds [10, 11]. It has been reported that GSH levels are naturally decreased over age [18]. The enzyme glutathione peroxidase 4 (GPx4) plays a crucial role in protecting cells against ferroptosis by removing toxic products of Fe-induced lipid peroxidation and preventing GSH depletion. A decrease in glutathione peroxidase 4 (GPx4) levels contributes to ferroptosis [15, 18].

The organism *Drosophila melanogaster* has made significant contributions to studies on the mechanisms that influence age-related functional declines. Furthermore, flies exhibit behavioral and biochemical similarities with vertebrates. Studies have shown that aging impairs the behavior, sensorimotor responses, reproductive behavior, and cognitive functions of flies [19], as well as age-related declines in physiological functions [20]. Additionally, the short life cycle of flies allows for the study of factors that could potentially interfere with life expectancy [21] and metal homeostasis [22–26].

In this study, we assessed behavioral and biochemical changes, oxidative damage, and the role of the antioxidant GSH in the damage induced by aging in *Drosophila melanogaster*.

## 2. Material and Methods

### 2.1. Chemicals

All the chemicals used in the study were purchased from Sigma–Aldrich e Labtest (São Paulo, SP, Brazil).

### 2.2. Drosophila Stock and Culture

*Drosophila melanogaster* (Harwich lineage) was kept under controlled temperature at 25° C and humidity at 60-70% in a light/dark cycle of 12 hours, cultivated in glass tubes measuring 50 mm × 85 mm and containing 10 mL of standard medium (corn flour, salt, wheat germ, powdered milk, sugar, soy flour, and rye flour) supplemented with dry yeast. Nipagin^®^ was used as an antifungal agent as described by Gomes et al. [18].

### 2.3. Experimental Design

**Figure 1:**
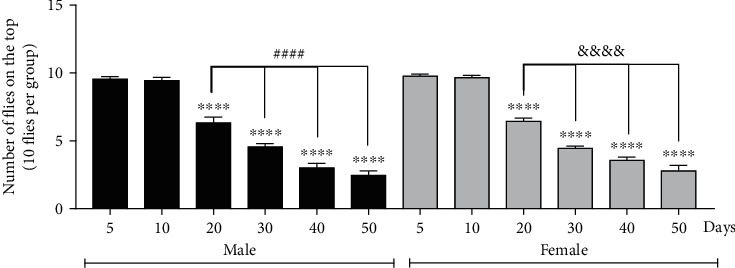
Negative geotaxis performed on the 5-, 10-, 20-, 30-, 40-, 50-day-old flies starts to show a decrease on the twentieth day of life for both sexes. The results are represented as mean ± standard error (SEM) and are expressed in number of flies on top. ^∗∗∗∗^*p* < 0.0001 about their respective control (5-day-old male or 5-day-old female), ^####^*p* < 0.0001 female in different old, and ^&&&&^*p* < 0.0001 male in different old.

**Figure 2:**
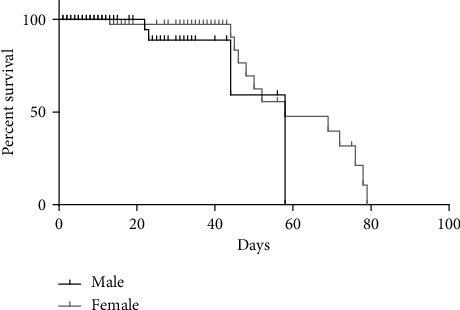
Female flies have a longer life expectancy than males. The black line refers to male survival, and the gray line refers to female survival.

The flies (3 days old) were isolated and separated by sex and kept in a standard medium for different periods for behavioral and biochemical analysis. The lifetime periods chosen for analysis were based on an analysis of motor performance over the aging of flies (Figure 1) and considering the lifespan of flies in laboratory conditions (Figure 2).

### 2.4. Lifespan Analysis

For lifespan analysis, 50 female flies and 50 male flies were placed separately per sex into vials containing 10 mL of standard food. Flies were transferred to fresh vials every 7 days. The number of dead flies was daily recorded.

### 2.5. Locomotor Analysis

#### 2.5.1. Negative Geotaxis

The negative geotaxis test (climbing ability) was performed in accordance with Bland et al. [19], with some modifications at 5 different ages (5, 10, 20, 30, 40, and 50 days of life). For this purpose, 10 flies per group were anesthetized in ice and placed in vertical glass tubes (length 25 cm, diameter 1.5 cm) closed with the respective lids. After 30 min of recovery, the flies were tapped lightly on the bottom of the tube, and the number of flies it took to rise 6 cm in the glass column was recorded. The test was repeated 3 times with intervals of 20 seconds each. Thirty flies were used per group (*n* = 30). All experiments were performed in triplicate. This experiment was carried out 9 times totalizing 90 individuals per group. Results were expressed as the number of flies on the top.

#### 2.5.2. Footbridge Test

To carry out the footbridge test, we used Solidworks software to design models and prototypes, which were then modeled in Blender. The resulting models were printed using acrylonitrile butadiene styrene (ABS) on a GTMax 3D Core A3 3D printer, following a protocol with a primary layer height of 0.1 mm. This protocol was based on Wosnitza et al. [21], with some modifications. The test consists of a 13 cm long and 0.5 cm wide horizontal footbridge (see Supplementary material I). The experimental groups included 5-day-old males, 5-day-old females, 30-day-old males, and 30-day-old females. The wings of the flies were clipped three hours before the tests to prevent flight. Males and females were analyzed separately, and individuals from each group crossed the footbridge test individually for up to 60 seconds. The time taken by flies to complete the path was recorded. Four groups of 20 flies of both sexes were analyzed. The results were expressed in seconds.

#### 2.5.3. Balance Test

To analyze the balance of flies at different life stages, we designed a platform using Solidworks software (Supplementary material II) that was 3 cm in length and printed on a 3D printer. This was done similarly to the previous catwalk test, following the method of Iliadi et al. [20] with modifications. The platform had two bases marking the beginning and the end of the walk, with a nylon thread (thickness 0.10 mm) crossing the platform. Flies of both sexes were divided into four groups of 20 individuals. The time taken by each fly to cross the line was evaluated for up to 60 seconds, and the time was recorded. The results were expressed in seconds.

### 2.6. Determination of Cellular Viability and Arbitrary Steady-State ROS Levels

A group of 20 flies 30-day-old (separated by sexes) and 20 flies with 5 days of life (separated by sexes) was homogenized in 1 mL of mitochondrial isolation buffer (220 mM mannitol, 68 mM sucrose, 10 mM KCl, 10 mM HEPES, and 1% BSA) and centrifugation at 1000 × g for 10 min (4°C). The supernatant enriched with mitochondria was used to determine cell viability (resazurin assay; 544 nm_ex_/590 nm_em_) and the ROS steady-state levels by using a fluorescent dye 2,7-dichlorofluorescein diacetate (DCF-DA; 485 nm_ex_/530 nm_em_). Assays were performed according to previously described protocols [18]. For the resazurin assay, 5 replicates were performed in triplicate, and for the DCF-DA assay, 4 replicates were performed in triplicate. Data were standardized by protein concentration and expressed in percentage.

### 2.7. Quantification of GSH Levels

GSH was measured according to Ellman [22] with minor modifications. 20 flies per group were homogenized in 1 mL of 0.5 M Tris/HCl pH 8.0 at 4°C. In the 96-well plate, 50 *μ*L of the supernatant, 190 *μ*L of the buffer used to homogenize, and 10 *μ*L of 5 mM DTNB were transferred and were left in the dark for 20 min, and the reading was performed at 412 nm in a microplate reader (PerkinElmer-EnSpire 2300 Multilabel Reader). The assay was repeated 7 times in triplicate.

### 2.8. Lipid Peroxidation

20 whole flies of different ages were anesthetized in ice and placed in vertical glass tubes (length 25 cm, diameter 1.5 cm) containing 600 *μ*L of TBA (0.005% in 20% trichloro acetic acid (TCA)) according to Sachett et al. [23], with some modifications (dilution of TBA from 0.5 to 0.005%). In this test, no homogenization was performed. The flies were incubated at 95°C for 60 minutes. The absorbance of samples was measured at 532 nm in a microplate reader (PerkinElmer-EnSpire 2300 Multilabel Reader). The results were expressed in absorbance, and the experiments were repeated 7 times in triplicate.

### 2.9. Quantification of Hemolymph Fe Levels

For the extraction of the hemolymph, microtubes of 1.5 mL and 0.5 mL were used. One was placed inside, and the smaller tube was perforated. Then, 40 flies were paralyzed on ice, and the thorax was perforated, and flies were placed into the 0.5 mL microtube and centrifuged at 3000 × g for 20 minutes to drain the hemolymph into a 1.5 mL tube. A volume of 1 *μ*L of hemolymph was obtained. This volume was diluted in 29 *μ*L of 10 mM phosphate-buffered saline (PBS) according to Herren et al. [24] and Masson et al. [25]. Fe levels were quantified in hemolymph using a colorimetric kit (Labtest^®^). The assay was based on the dissociation of ferric ions (Fe^3+^) from transferrin by the action of an acidic pH buffer and reduced to ferrous ions (Fe^2+^) by the action of hydroxylamine. Upon the addition of ferrozine, a bright magenta complex was formed, and absorbance was measured at 540 and 580 nm. The Fe levels were expressed as mg/dL of hemolymph. The assay was performed 5 times in triplicate.

### 2.10. Diethyl Maleate (DEM) Treatment and Analysis

To investigate the involvement of GSH in locomotor deficits associated with aging, it used diethyl maleate (DEM), a GSH depletor that complexes with GSH and decreases the levels of this antioxidant. 50 flies per group with 5- and 30-day-old male and female flies were exposed to 1 mM DEM mixed with the medium for 24 h. After, the negative geotaxis test and GSH levels were evaluated as described above [26].

### 2.11. Statistical Analysis

All data were submitted to the normality test D'Agostino and Pearson, Shapiro-Wilk, and Kolmogorov-Smirnov. The parametric data were expressed as mean ± standard error mean (SEM) and were analyzed by two-way ANOVA followed by Tukey's *post hoc* test; and nonparametric data were expressed as a median interquartile range, analyzed by Kruskal-Wallis followed by Dunn's *post hoc* test. The results were considered statistically significant when *p* ≤ 0.05.

## 3. Results

### 3.1. Differences in the Lifespan of Male and Female Flies

We performed a survival curve to analyze the difference in life expectancy between males and females. The curve indicated that the life expectancy of females was longer than that of males (by approximately 20 days) (Figure 2).

### 3.2. Aging Impairs Negative Geotaxis of Flies

To assess whether the climbing ability is affected by age, we performed the negative geotaxis test on male and female flies of different ages. As shown in Figure 1, locomotor deficits were observed in both male and female flies after 20 days of life, and these deficits increased with age.

### 3.3. Footbridge Test Showed That Aging Flies Have a Lower Running than Younger Flies

**Figure 3:**
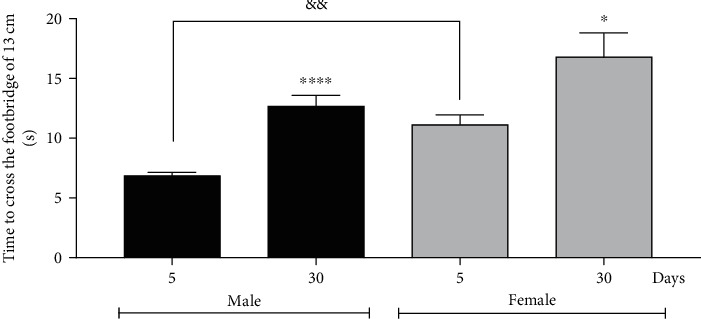
Males and females of *Drosophila melanogaster* with 30-day-old show a decrease in walking ability performed by the footbridge test. In addition, 5-day-old males are faster than females of the same age. The results are represented as mean ± standard error (SEM) and are expressed in time to cross. ^∗^*p* < 0.05 and ^∗∗∗∗^*p* < 0.0001 in relation to their respective control (5-day-old male or 5 day-old-female) and ^&&^*p* < 0.01, in relation to 5-day-old males with 5-day-old females.

Taking into account the compromised climbing ability with age, we also analyzed other parameters such as walking speed and balance. The walking speed was evaluated in the footbridge test through a horizontal 13 cm long and 0.5 cm wide apparatus printed on a 3D printer (supplementary material I and Figure 3). The group of 5-day-old males took an average of 6 seconds to cross the 13 cm footbridge, while 5-day-old females took an average of 11 seconds. On the other hand, 30-day-old males took an average of 12 seconds, and 30-day-old females took an average of 17 seconds.

### 3.4. The Fly's Balance Is Affected by Aging

**Figure 4:**
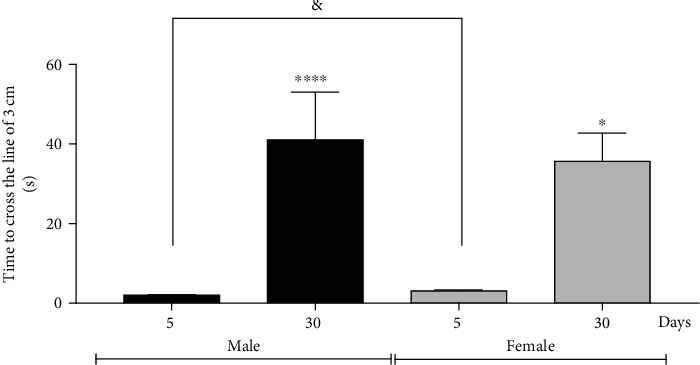
Males and females of *Drosophila melanogaster* with 30-day-old show a drop in the balance test. Males at 5-day-old cross the 3 cm line more easily than females of the same age. The graph represents the taken-to-crosse a 3 cm line. The results are represented as SEM. ^∗^*p* < 0.05 and ^∗∗∗^*p* < 0.0001 and in relation to their respective control (5-day-old male or 5-day-old female); ^&^*p* < 0.05 in relation to a 5-day-old male with 5-day-old female.

Considering the detrimental effects of age on the locomotor ability of flies, it evaluated the balance of young and old flies in both sexes. This test was conducted on a 3 cm long platform, where flies navigated across a continuous nylon wire (with a thickness of 0.10 mm) that spanned from the beginning to the end of the platform. The model was printed on a 3D printer using Solidworks software and can be visualized in Supplementary Material II. The group of 5-day-old males takes an average of 2 seconds to cross the 3 cm line, while the 5-day-old females take an average of 3 seconds, and the group of 30-day-old males takes an average of 40 seconds to cross the line, and 30-day-old females take an average of 35 seconds (Figure 4).

#### 3.4.1. Age Decreases Cell Viability and Induces ROS in Flies

**Figure 5:**
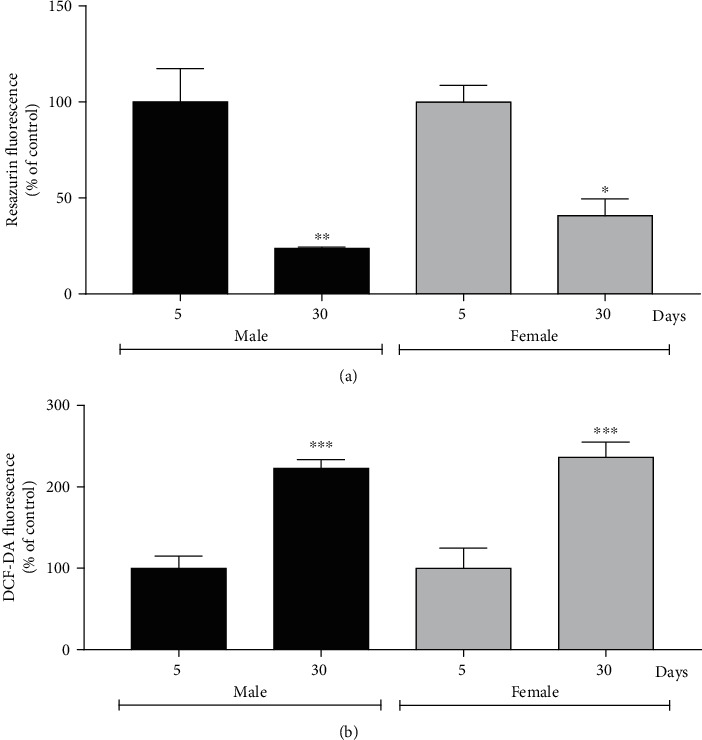
Males and females of *Drosophila melanogaster* at 30-day-old show a decrease in cell viability and increased levels of ROS. (a) Cell viability and (b) ROS levels. The results are represented as mean ± standard error (SEM) and are expressed as a percentage. ^∗^*p* < 0.05, ^∗∗^*p* < 0.01, and ^∗∗∗∗^*p* < 0.0001 in relation to their respective control (5-day-old male or 5-day-old female).

After observing changes in behavioral assays, biochemical parameters associated with the presence of ferroptosis, including cell viability and ROS levels in young and old flies of both sexes, were analyzed. The 30-day-old male group showed a 4.3 times decline in mitochondrial dehydrogenase activity compared to the 5-day-old male group, and the 30-day-old female group showed a 2.5 times decline relative to the 5-day-old female group, represented by the fluorescence of the compound resazurin (Figure 5(a)). The DCF-DA fluorescence for detecting ROS was increased by 2.2 times in the group of 30-day-old males and 30-day-old females by 2.3 times about their respective controls (5-day-old flies). It was observed that 30-day-old males decreased cell viability by 2.6 times in comparison with 30-day-old females, whereas no differences between these groups were observed in the DCF-DA test (Figure 5(b)).

### 3.5. GSH Levels Are Decreased in Aging Flies

**Figure 6:**
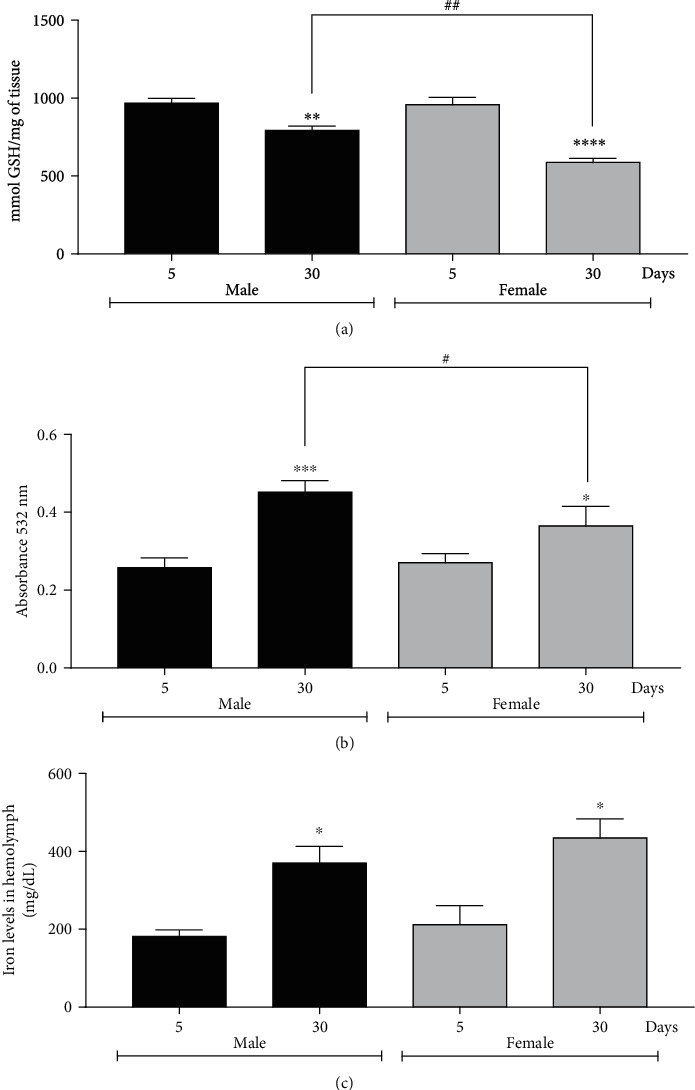
Males and females of *Drosophila melanogaster* at 30-day-old show a decrease in GSH levels, an increase in lipid peroxidation, and an increase in Fe levels. In addition, males and females with 30 days of life differed from each other in GSH assay and lipid peroxidation assay. (a) GSH levels, (b) lipid peroxidation, and (c) Fe levels. The results are represented as mean ± standard error (SEM) and expressed as follows: (a) mmol GSH/mg of tissue, (b) Tbars absorbance, and (c) mg/dL of hemolymph ^∗^*p* < 0.05, ^∗∗^*p* < 0.01, ^∗∗∗^*p* < 0.001, and ^∗∗∗∗^*p* < 0.0001 about their respective control (5-day-old male or 5-day-old female) and #*p* < 0.05 and ## *p* < 0.01 in relation the 30-day-old male with 30-day-old female.

A hallmark of ferroptosis is a decrease in GSH levels; thus, this parameter was analyzed in young and old flies of both sexes. In the group of males with 30-day-old, in a decrease of 1.2 times about the group of 5-day-old males and in 30-day-old females, a decrease of 1.6 times about their respective controls (5-day-old females) was observed. Moreover, 30-day-old males have 1.3 times more GSH than 30-day-old females (Figure 6(a)).

### 3.6. Age Induces Lipid Peroxidation in Male and Female Aging Flies

Considering the increase in lipid peroxidation and lower levels of GSH, indicating ferroptosis, induction of lipid peroxidation was investigated. In this trial, we observed an increase of 2 times and in the group of 30-day-old females an increase of 1.5 times, both compared to their respective controls (5-day-old males and females), and when comparing 30-day-old males and females, it was observed that males present a 1.3 times increase in lipid peroxidation (Figure 6(b)).

### 3.7. Age Increases Fe Levels in Fly's Hemolymph

Ferroptosis is an Fe-dependent cell death; thus, the levels of this metal were evaluated in the hemolymph of flies. Fe amount was evaluated in young and old flies of both sexes. It observed a 2 times increase in Fe content in 30-day-old male and female flies in comparison with their respective controls (5-day-old males and 5-day-old females) (Figure 6(c)).

### 3.8. Diethyl Maleate Treatment Causes Mortality and Decreases GSH Levels and Locomotor Performance in *D. melanogaster*

**Figure 7:**
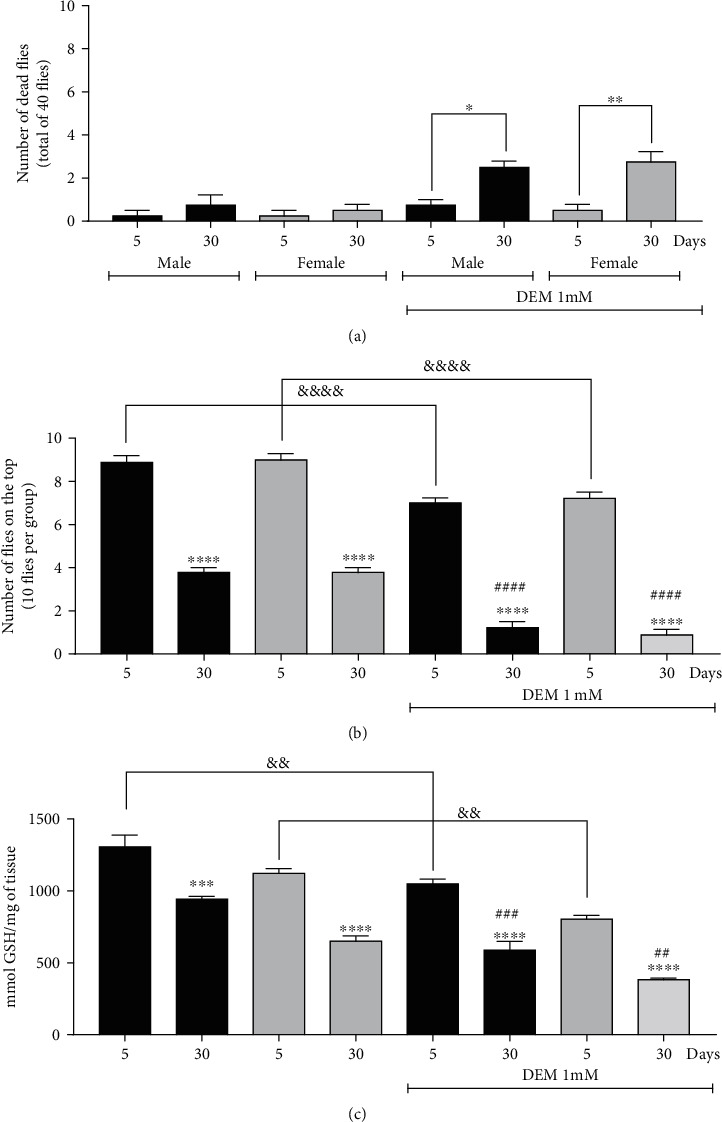
GSH depletor (DEM) decreases GSH levels and negative geotaxis in 30-day-old males and females of Drosophila melanogaster. (a) Mortality caused by DEM after 24 h of exposure, (b) negative geotaxis, and (c) GSH levels. Results are represented as error (SEM) and are expressed (a) in number of dead flies, (b) number of flies on the top, and (c) per mmol GSH/mg of tissue. ^∗^*p* < 0.05, ^∗∗^*p* < 0.01, ^∗∗∗^*p* < 0.001, and ^∗∗∗^*p* < 0.0001 about their respective control (5-day-old male or 5-day-old female) and ##*p* < 0.01, ###*p* < 0.001, and ####*p* < 0.0001 in relation to the same group but without DEM and ^&&^*p* < 0.01 and ^&&&&^*p* < 0.0001 about 5-day-old male with 5-day-old female with and without DEM.

GSH prevents ferroptosis by blocking lipid peroxidation or by acting as a ligand for Fe in the cytoplasm [13]. To investigate the involvement of GSH in the deleterious effects of aging on behavior and mortality, flies were exposed to DEM, a GSH depletor. The results showed that the DEM induced a decrease in the locomotor test of 3 times and 4.6 times in aged males and females about the control (without DEM). Furthermore, a 1.5 times and 1.6 times decrease in GSH levels was observed in male and female aging flies (30 days) exposed to DEM compared to their respective controls (without DEM). Male 5-day-old flies presented a 1.2 times decrease in locomotor performance in the negative geotaxis test when compared to the group that was not exposed to DEM. Similar results were observed in females. Regarding GSH levels, a 1.2 times decrease was observed in 5-day-old males and a 1.3 times decrease in 5-day-old females, both compared to their respective controls (5-day-old males and 5-day-old females without DEM) (Figure 7). The treatment with DEM induced a 2.5 times increase in the mortality of male and female aging flies.

## 4. Discussion

Aging comprises natural physiological changes that occur in the organisms over time including progressive decline of locomotor and cognitive functions. Multiple events from molecular levels to organ systems mediate the aging process. The cumulative damage caused by reactive oxygen species (ROS) comprises one of the main theories to explain aging [27, 28], and more recently, an excess of Fe has been involved in the progression of aging effects, by the promotion of a type of oxidative cell death called ferroptosis [6].

In the present study, it investigated the detrimental effects of age on the behavior of males and females of *Drosophila melanogaster* and the involvement of ferroptosis in this process by evaluating mortality, behavior alterations, lipid peroxidation, ROS production, and GSH levels. Locomotor deficits in *D. melanogaster* were evaluated in different ages by the analysis of negative geotaxis, footbridge test, and balance test. Our results demonstrated detrimental effects of aging on locomotor behavior after 20 days of life that progressed over aging equally in both sexes. Our findings are in accordance with previous studies which reported a decline in locomotion of aging Drosophilas [29, 30]. Moreover, for aging flies, the time to cross the footbridge and the line by flies were longer than younger flies, and many aging flies were unable to perform the balance test and were not included in the analysis. In this study, younger male flies display better performance in locomotor tests. Videlier et al. [31] demonstrated that locomotor activity in Drosophila is influenced by body mass, reproductive status, and sex, among other factors, and males are more active than females in daylight peaks. This behavior might reflect an increase in the energetic investment of males in the behavior of mate acquisition, thus increasing mating frequency and reproductive success [32]. In contrast, both male and female flies' performance in the tests was affected by age. Locomotor ability depends on a combined activity of the nervous system and musculature. Thus, a decline in locomotor performance observed in aging flies might result from a defect in both systems associated with cumulative damage caused by ROS [29, 33, 34], thus possibly decreasing the metabolic viability of cells as observed in this study.

The role of Fe in the progress of aging has been the focus of attention, and an imbalance of Fe metabolism is more prevalent in the elderly population leading to detrimental consequences and increasing the risk of mortality [10]. An excess of Fe can deflagrate ferroptosis, which consists of a nonapoptotic mechanism of cell death, characterized by massive damage to cells, and has been implicated in neurological and cardiovascular diseases and cancers [17]. In this study, it was investigated for the first time the contribution of ferroptosis to detrimental effects of aging in fruit flies, in males and females. An augmented level of Fe was observed in aging fly hemolymph. An accumulation of Fe has been demonstrated to be common in different species, as demonstrated in *C. elegans* and human brain. The mechanism possibly implied in this phenomenon is a compromised capacity of ferritin to store Fe and imbalance favoring Fe influx. It is known that redox-active metals, like Fe, are kept at low concentrations (0.2-0.5 *μ*M) under physiological conditions, and the excess is bound to proteins, including ferritin, avoiding toxic responses [35]. In this study, GSH level was decreased in aging flies of both sexes, and a more prominent decrease was observed in females. GSH is an endogenous antioxidant acting on the neutralization of free radicals including °OH and is a reduced cofactor of glutathione peroxidase 4 (GPx4) [11], and in the cytosol, GSH acts as an Fe (II) ligand [36]. A decrease in GSH could contribute to ROS accumulation and lead to Fe^2+^ release from ligand proteins contributing to the Fenton reaction, where Fe catalyzes the H_2_O_2_ breakdown yielding hydroxyl radicals and causing lipid peroxidation, a vital hallmark of ferroptosis [12]. In this regard, previous studies investigated comparative levels of GSH over aging in male and female of rats. It was observed that GSH content changed differently between tissues analyzed comparing males and females over aging [37] The decrease in GSH in aging has been related to a reduction in de novo glutathione synthesis, as evidenced by a decrease in the levels of protein *γ*-glutamylcysteine synthetase (GCS) in aging rat brain. Thus, our study does not provide enough results to explain the more prominent decrease in GSH in aging female flies; however, a hypothesis of a differential regulation in GSH synthesis of males and females over time and a tissue-specific decrease in GSH between sexes should not be discarded. Additionally, our study demonstrated that females presented a higher life expectancy than males. This increase could not be attributed uniquely to the GSH levels, once a similar amount of GSH was found in males and females. It is important to mention that in other studies, similarly, females tend to live significantly longer than males. These phenomena have been attributed to multifactorial factors including genetic, epigenetic, and environmental factors [38].

To understand the contribution of GSH decrease for detrimental effects of age, GSH levels were decreased with DEM. The results demonstrated a significant decrease in GSH by treatment with DEM in young and aging flies. Moreover, augmented mortality and a decrease in locomotor performance were observed in aging and young flies. These findings highlight the protective role of GSH against oxidative damage induced by augmented levels of Fe in flies of both sexes and evidence the contribution of ferroptosis to the detrimental effects of age. Similarly, it has been earlier demonstrated in increased Fe levels and free radical production, in parallel with a decrease in GSH in aging *C. elegans* [6, 26, 34].

**Figure 8:**
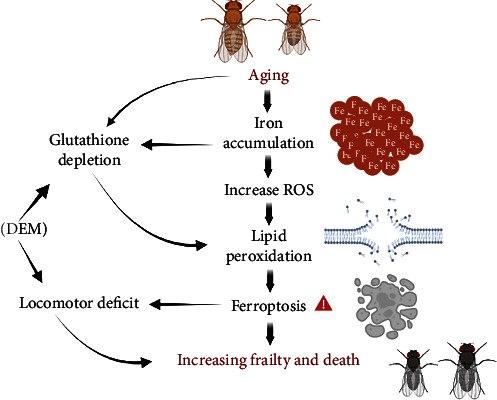
Proposed hypothesis for Fe-induced damage in aging. Natural aging leads to Fe accumulation and glutathione depletion, this Fe accumulation increases ROS, leading to lipid peroxidation, and glutathione depletion also leads to this damage, occurring ferroptosis that causes changes in behaviors and ultimately fragility and death. DEM worsens locomotor damage and accelerates death.

Increasing evidence suggests that sex and gender play a significant role in the etiology, presentation, and treatment of various diseases. Important discoveries regarding the implication of sex and gender in diseases have been made through research on the organism *Drosophila melanogaster*. For instance, previous studies have reported differential responses between sexes in Drosophila concerning inflammatory response and biotransformation of toxic substances [39, 40]. Our group's previous research has reported differential susceptibility of males and females to 1-octen-3-ol (volatile organic compound) [41]. In this study, the comparative analysis between sexes in Drosophila provides important insights into the differential effects of aging on behavioral and biochemical parameters in males and females. These findings could underscore the importance of extending studies and considering sex in precision medicine approaches. A proposed hypothesis for Fe-induced damage in aging is represented in Figure 8.

## 5. Conclusion

The present study investigated the effects of aging on lifespan, oxidative damage, behavioral alterations, and iron (Fe) levels in male and female *Drosophila melanogaster* and correlated these parameters with ferroptosis-related processes. Results showed that aging led to GSH depletion, Fe accumulation, increased reactive oxygen species (ROS) production, and lipid peroxidation. The study highlighted the protective role of GSH against aging-induced damage in flies. Overall, these findings provide valuable insights into the involvement of ferroptosis in the aging process of *D. melanogaster* and suggest that this organism may serve as a reliable model for investigating aging-related mechanisms.

## Data Availability

Data will be made available upon request.

## Supplementary Materials

Supplementary 1 Supplementary material I: footbridge test. Model made in Solidworks software and printed on a 3D printer. The horizontal footbridge was 13 cm long and 0.5 cm wide.

Supplementary 2 Supplementary material II: balance test. Model made in Solidworks software and printed on a 3D printer. Platform is 3 cm long with two bases marking the beginning and end of the walk. A nylon wire (thickness 0.10 mm) passes through this platform and is connected to the starting bases.
