# Supplementary figures and images for: Increased Iron Levels and Oxidative Stress Mediate Age-Related Impairments in Male and Female Drosophila melanogaster
Source: Oxid Med Cell Longev. 2023 Jun 9;2023:7222462. doi: 10.1155/2023/7222462 (PMC10275690; doi:10.1155/2023/7222462)

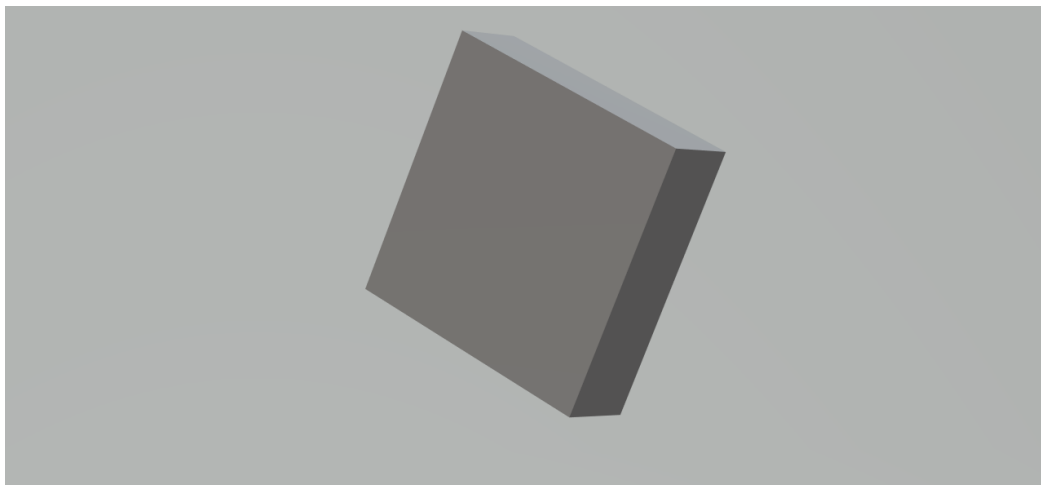

Supplementary material I

Supplement: Supplementary 1 — Supplementary material I: footbridge test. Model made in Solidworks software and printed on a 3D printer. The horizontal footbridge was 13 cm long and 0.5 cm wide. [file 7222462.f1.pdf]

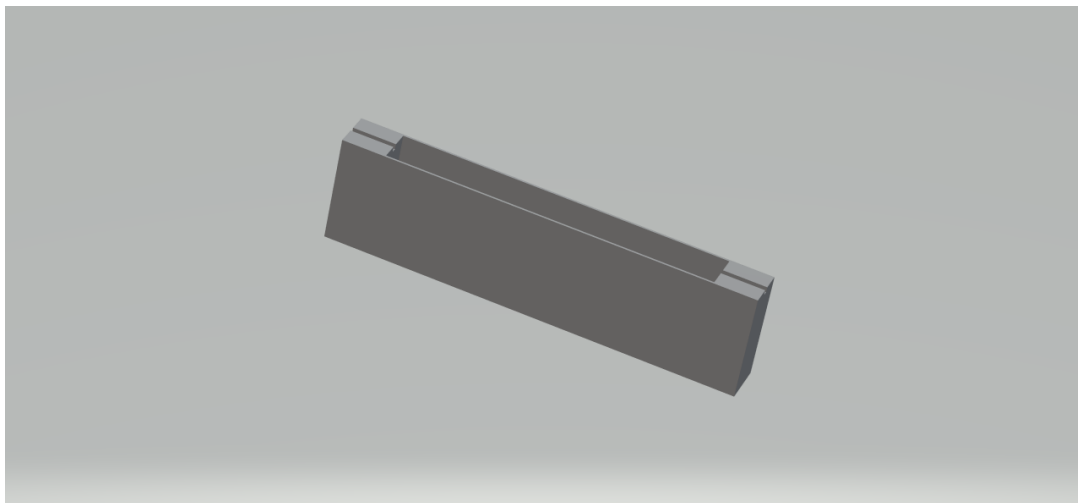

Supplementary material II

Supplement: Supplementary 2 — Supplementary material II: balance test. Model made in Solidworks software and printed on a 3D printer. Platform is 3 cm long with two bases marking the beginning and end of the walk. A nylon wire (thickness 0.10 mm) passes through this platform and is connected to the starting bases. [file 7222462.f2.pdf]
